# The selective anti-proliferative and pro-apoptotic effect of *A. cherimola* on MDA-MB-231 breast cancer cell line

**DOI:** 10.1186/s12906-020-03120-1

**Published:** 2020-11-13

**Authors:** Maria Younes, Carl Ammoury, Tony Haykal, Leah Nasr, Rita Sarkis, Sandra Rizk

**Affiliations:** 1 Department of Natural Sciences, Lebanese American University, Byblos, Lebanon; 2 Laboratory of Regenerative Hematopoiesis, Swiss Institute for Experimental Cancer Research (ISREC) & Institute of Bioengineering (IBI), School of Life Sciences, Ecole Polytechnique Fédérale de Lausanne (EPFL), 1015 Lausanne, Switzerland

**Keywords:** *Annona cherimola*, Apoptosis, Breast Cancer, MDA-MB-231, Medicinal foods

## Abstract

**Background:**

Herbal medicines have been a major target for numerous studies through the past years as an alternative treatment for cancer, mainly due to their minimal effects on normal healthy cells. *Annona cherimola,* popularly known as Cherimoya, is an edible natural fruit rich in phytochemical components and known to possess various biological activities. Previous studies have reported the anti-cancerous effect of *A. cherimola* ethanolic leaf extract (AELE) on leukemia. This study aims at studying the potential anti-cancer activity of this extract in vitro in two different breast cancer cell lines, namely MDA-MB-231 and MCF-7, in addition to investigating its toxicity on normal mesenchymal stem cells.

**Methods:**

The anti-proliferative effect of AELE was evaluated via cell viability assay. Propidium iodide staining, Cell Death Detection ELISA and flow cytometry analysis of Annexin V binding were used to assess cell cycle progression, DNA fragmentation and apoptosis induction, respectively. Protein expression was determined via Western Blot analysis to decipher the underlying apoptotic molecular mechanism induced upon AELE treatment.

**Results:**

The anti-proliferative effect of the extract was found to be selective on the triple-negative breast cancer cell line (MDA-MB-231) in a time- and dose-dependent manner with an IC_50_ of 390.2 μg/mL at 48 h, with no cytotoxic effects on normal murine mesenchymal stem cells. The pro-apoptotic effect was confirmed by the increase in cellular and DNA fragmentation, flipping of the phosphatidylserine moiety to the outer leaflet, and the increase in Annexin V binding. The underlying molecular mechanism revealed the involvement of the mitochondrial pathway, as shown by alterations in mitochondrial permeability and the upregulation of cytochrome c expression.

**Conclusion:**

All the data presented in our study suggest that AELE exhibits a selective anti-proliferative and pro-apoptotic effect on the chemo-resistant MDA-MB-231 breast cancer cells, providing evidence for the anti-tumor effects of *A. cherimola*.

## Background

In recent years, functional foods have been of a major interest for research, not only for their nutritional values, but also for their physiological and biological activities [1]. Plant-derived products have major contributions in the medical field, particularly for the identification of novel drugs [2]. Medicinal plants, effective in treating various diseases including Alzheimer’s [3], malaria [4], cancer [5], and microbial infections [6] have been extensively investigated during the past decades.

Annonaceae is a large family of tropical plants commonly known as custard apple that has been considerably studied [7, 8]. Phytochemical analysis of the plant showed that it is rich in alkaloids, terpenoids, flavonoids, and acetogenins [9]. Moreover, it is one of the most genus-rich and species-rich family, characterized by the high morphological diversity of its species [10]. *Annona* is one of the 129 genera of Annonaceae, which includes 119 species [11] that vary based on their origin, climate, and topography [9]. *Annona* species were excessively used in alternative medicine because of their antimalarial [4], anti-parasitic [12], anti-inflammatory [13], and anti-cancerous [11] properties. The flavonoids and acetogenins found in the leaves of *A. muricata* Linn, also known as Graviola, were found to exhibit an antiproliferative effect on prostate cancer [14] and induce apoptosis in vitro and in vivo on breast cancer cell lines [15]. Likewise, *A. squamosa* seed extract displayed a cytotoxic effect on leukemic and breast cancer cell lines in vitro through oxidative stress and downregulation of Bcl-2 [16].

*Annona cherimola* Mill also known as Cherimoya, is an edible subtropical fruit with a sweet flavor containing nutritional components such as vitamins and minerals [17]. On the other hand, *A. cherimola* was greatly used for various applications in digestive disorder and skin disorders [18]. Studies conducted on the seeds of Cherimoya revealed their potential to induce apoptosis in Acute myeloid leukemia (AML) cell lines through the intrinsic and extrinsic pathways [19], and to exhibit a cytotoxic effect on prostate cancer cell line due to the presence of annomolin and annocherimolin [20]. Similarly, the terpene-rich ethanolic leaf extract of *A. cherimola* displayed an anti-proliferative and a pro-apoptotic effect on AML cell lines [21].

Breast cancer is considered the most frequently diagnosed cancer in women and a leading cause of death worldwide [22]. It is a heterogeneous disease with several subtypes differing in their clinical and histopathological features. Breast cancer is defined either as non-invasive, starting in the ducts or lobules of the breast without spreading to other healthy breast tissues or invasive, spreading to breast, lymph, or distant healthy tissues [23]. Furthermore, it is classified into different groups, depending on the expression of different receptors i.e., estrogen receptor (ER), progesterone receptor (PR), and human epidermal growth factor receptor 2 [24]. Based on these classifications and other clinical factors, breast cancer is treated by surgery, radiation therapy, chemotherapy, or hormone therapy [22]. However, these strategies are responsible for major side effects and require an alternative therapy, effective in treating cancerous cells without harming normal cells [25].

This study investigates the potential anti-cancer effect of the ethanolic leaf extract of *A. cherimola* on breast cancer cell lines in vitro.

## Methods

### Breast cancer cell culture

Two breast cancer cell lines were obtained from American Type Culture Collection: the triple-negative Breast Cancer Cell line MDA-MB-231, isolated from a pleural effusion of a patient with invasive ductal carcinoma and used to model late-stage breast cancer [26], and the estrogen-dependent MCF-7 cell line, which is an adherent epithelial luminal cell line positive for ER and PR [27]. The cells were cultured (37 °C, 5% CO_2_) in Dulbecco’s Modified Eagle Medium (DMEM, Sigma-Aldrich) supplemented with 10% Fetal Bovine Serum (FBS, Gibco™) and antibiotics (100 U/mL penicillin and 100 μg/mL streptomycin from Pen-Strep Lonza) [28]. The cell lines were split as previously mentioned by Khalife et al. [29].

### Isolation and culture of Mesenchymal stem cells (MSCs) from rat bone marrow

MSCs were isolated from rat bone marrow as previously described [19]. All experiments were approved by the University’s Animal Care and Use Committee and complied with the Guide for the Care and Use of Laboratory Animals [30, 31]. Briefly, the bone marrows from tibial and femoral bones were flushed, and the collected cells were incubated. After 5 days, MSCs were identified by their spindle-shaped morphology [32].

### Plant material and preparation of crude leaf extracts

*Annona cherimola* leaves were obtained from a tree located in Awkar-Lebanon (90 m Above Sea Level), in January 2018, and identified by Dr. Nisrine Machaka-Houri. A voucher specimen was deposited in Beirut Arab University Herbarium (RCED2019–362). 91.3 g of leaves were ground, shaken and the ethanolic extract (AELE) was prepared as previously described [21].

### Cell viability assay

MDA-MB-231 and MCF-7 cells were cultured in 96-well plates in triplicates (density = 1.5 × 10^5^ cells/mL), and were incubated overnight before treatment with, 174, 261, 348, 522, and 696 μg/mL of AELE for 24 h and 48 h. Topotecan (Abcam, 20 μM) [33, 34] and cisplatin (Mylan, 30 μM) [35, 36] were used as positive controls. MTS cell viability reagent (Promega) was used to assess the effect of AELE on the cell lines as detailed by Khalil [37] . Metabolically active cells were quantified by measuring the absorbance of each well at 492 nm, using Varioskan™ LUX multimode microplate reader. Percentage proliferation was calculated by dividing the absorbance of the treated cells with the average absorbance of the control untreated cells. IC_50_ values were calculated using GraphPad Prism 8.

### Cell cycle analysis

MDA-MB-231 cells (1.5 × 10^5^ cells/mL) were cultured in 6-well plates and incubated overnight. After treatment for 24 h with 261 and 522 μg/mL of AELE and 20 μM topotecan (positive control), the cells were stained with Propidium iodide (PI, Abcam, Cambridge, UK) following fixation with ice-cold absolute ethanol [38]. The Accuri C6 flow cytometer was used to assess their DNA content and were classified as follows: sub-G0/G1 phase cells (Pre-G or dead cells) have <2n, G0/G1 phase cells have 2n, S phase cells have between 2n and 4n, and G2/M phase cells have 4n.

### Cell death detection ELISA

MDA-MB-231 cells (1.5 × 10^5^ cells/mL) were seeded in 6 well plates and incubated overnight. Two different concentrations of AELE (261 and 522 μg/mL) were then added to the cells since these are the closest to the IC_50_ reported in the cell viability assay. Topotecan (20 μM) was used as a positive control. After 24 h, cells were extracted and lysed with incubation buffer using the Cell Death ELISA kit (Roche). Fragmented cytosolic nucleosomes were then isolated, and the procedure was completed as previously described [39].

### Apoptosis detection using fluorescent Annexin V staining

MDA-MB-231 cells were cultured and treated with AELE (261 and 522 μg/mL) and topotecan (20 μM) as described in previous sections. After 24 h, cells were stained with Annexin-V-FITC (Abcam, Cambridge, UK) and visualized under the bright-field conditions and FITC set filters with the ZOE fluorescent cell imager. The obtained images were then merged. Annexin V binding to the membrane reflects the translocation of the phosphatidylserine moieties which is a hallmark of apoptosis.

### Apoptosis quantification using flow cytometry

MDA-MB-231 cells were cultured and treated as described in the previous sections. After incubation for 24 h with AELE (261 and 522 μg/mL) and topotecan (20 μM), samples were collected, centrifuged at 1500 rpm and 4 °C, resuspended in suspension buffer, and then stained with Annexin V-FITC (Annexin V–fluorescein isothiocyanate [FITC] Apoptosis Detection Kit, Abcam) according to the manufacturer’s instructions. Samples were then analyzed using the Guava easyCyte™ flow cytometer [40].

### Western blot

Petri dishes were used to culture and treat MDA-MB-231 cells (2 × 10^5^ cells/mL) with AELE (261 and 522 μg/mL) for 24 h. The Qproteome mammalian protein prep kit (Qiagen, Hildren, Germany) was used to extract the proteins, which were then quantified based on the Lowry method. Proteins were prepared for separation by Sodium Dodecyl Sulfate-Polyacrylamide Gel Electrophoresis (10%) as explained by El Zein et al. [41], and transferred to Polyvinylidene fluoride membranes for protein assessment, as previously described [42]. Membranes were incubated with primary antibodies (1:1000) anti-cytochrome c (Abcam, Cambridge, UK), anti-p21 (Cell Signaling, Danvers, MA), anti-Bax and anti-Bcl2 (Elabscience, Houston, TX, USA). The internal loading control was detected using anti-β-actin (Santa Cruz Biotechnology, Dallas, TX, USA). After washing, the secondary antibody (Bio-Rad, Hercules, CA, USA) was added at the recommended concentrations (2:5000). Blots were visualized on ChemiDoc machine (BioRad, Hercules, CA, USA) and relative expression of proteins bands was quantified using the ImageJ program [43].

### Statistical analysis

All experiments were carried out in triplicates and repeated at least three independent times. The values were reported as mean ± SEM. The post hoc test used for the statistical analysis was Dunnett’s method to compare the different concentrations to the control and Sidak’s test was used to compare different time points of each concentration. *P*-values were calculated by t-tests or two-way ANOVA depending on the experiment, using GraphPad Prism 8. Significant differences were reported with * indicating a *p*-value: 0.01 < *p* < 0.05, ** indicating a p-value: 0.001 < *p* < 0.01, *** indicating a p-value: *p* < 0.001.

## Results

### The selective effect of *A. cherimola* ethanolic leaf extracts on the breast cancer cells proliferation

**Fig. 1:**
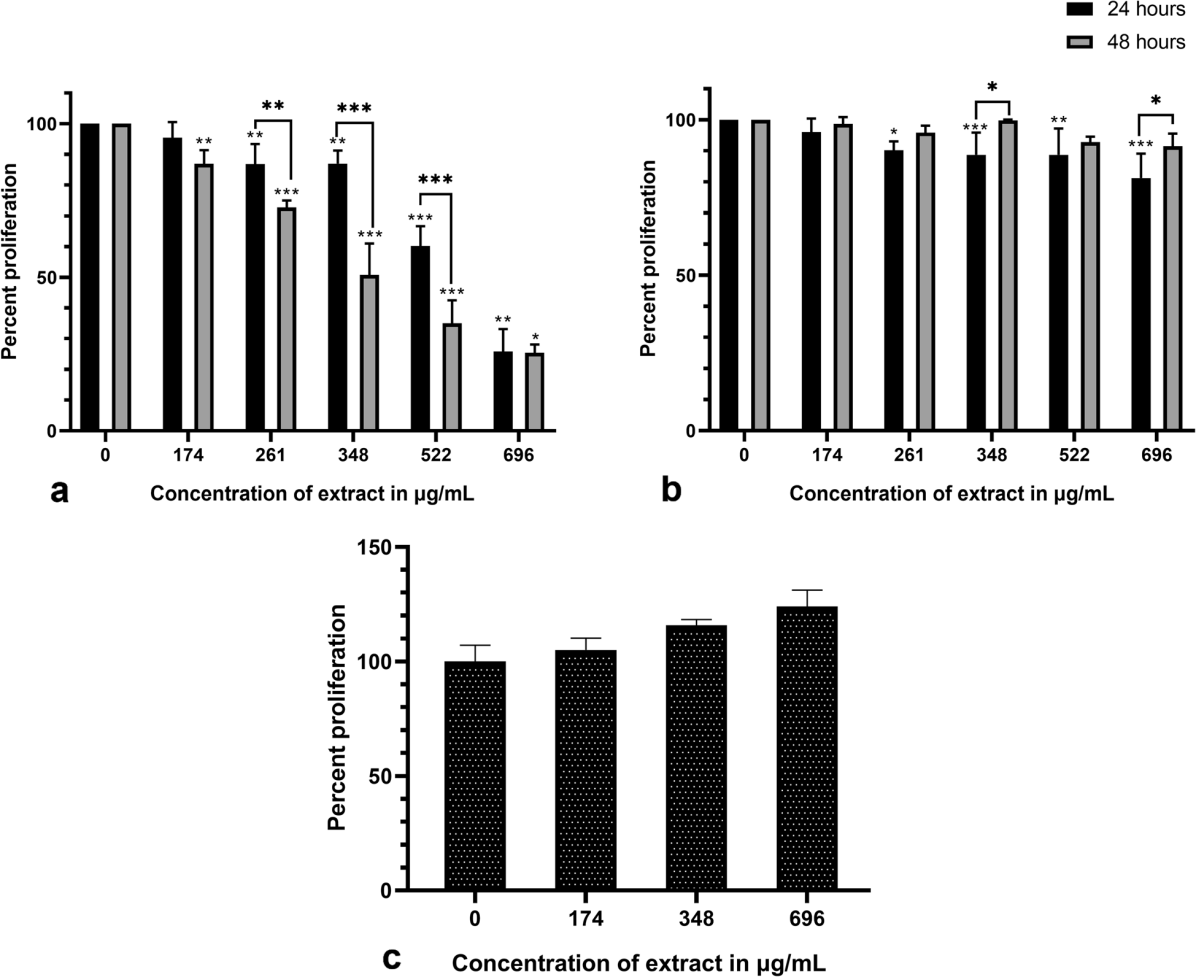
The selective effect of AELE on breast cancer cell proliferation using MTS assay. Percent proliferation of cells upon increasing AELE treatment. Bar graph showing the percent proliferation of MDA-MB-231 (**a**), MCF-7 (**b**) and Mesenchymal Stem Cells (MSCs) (**c**) treated with AELE. AELE showed a significant dose- and time- dependent inhibition of MDA-MB-231 cell proliferation and no inhibitory effect on MSCs isolated from rat bone marrow upon increasing AELE concentration. Dunnett’s test was used to compare the different concentrations to the control, and Sidak’s test was used to compare different time points of each concentration. Two-way ANOVA was used to determine the significant differences reported with * indicating a *p*-value: 0.01 < *p* < 0.05, ** indicating a *p*- value: 0.001 < *p* < 0.01, and *** indicating a p-value: *p* < 0.001

The effect of AELE on the proliferation of MDA-MB-231, MCF-7, and MSCs was quantified using the viability reagent MTS. A dose and time-dependent significant decrease in the proliferation of MDA-MB-231 was observed with a half-maximal inhibitory concentration (IC_50_) of 555.3 μg/mL and 390.2 μg/mL (Fig. 1.a) at 24 and 48 h post-AELE treatment, respectively. The viability was significantly reduced to less than 50% at higher treatment doses. The maximum concentration of treatment used (696 μg/mL) exhibited a percentage proliferation of 25.86 and 25.48% for MDA-MB-231, after 24 h and 48 h, respectively. A minimal effect was observed on MCF-7 cells, whereby the IC_50_ was not reached, and the maximal AELE concentration used (696 μg/mL) exhibited a percentage proliferation of 81.18 and 91.50% (Fig. 1.b), after 24 h and 48 h respectively, suggesting a selective anti-proliferative effect of AELE on MDA-MB-231 but not MCF-7. Treatment with 20 μM of topotecan exhibited a percentage proliferation of 59.61, 36.31% on MDA-MB-231 cells, 31.03, 19.36% on MCF-7 cells at 24 and 48 h, respectively. Similarly, treatment with 30 μM of cisplatin exhibited a percentage proliferation of 56.32, 32.57% on MDA-MB-231 cells, 46.78, 17.53% on MCF-7 at 24 and 48 h, respectively. The remaining experiments were therefore performed on MDA-MB-231, using the two concentrations closest to the IC50 (261 and 522 μg/mL), in addition to topotecan as a positive control.

To check for the specificity of the effects observed on MDA-MB-231 cells, normal MSCs from rat bone marrow were treated with AELE under similar conditions. Interestingly, the extract had no significant cytotoxic effect on normal cells even at the highest concentrations used (Fig. 1.c). It can be inferred that AELE exhibited selective anti-proliferative effects on MDA-MB-231, but not on normal cells.

### A cherimola leaf extract induces cellular fragmentation and an increase in pre-G0/G1 cells in MDA-MB-231 cells

**Fig. 2:**
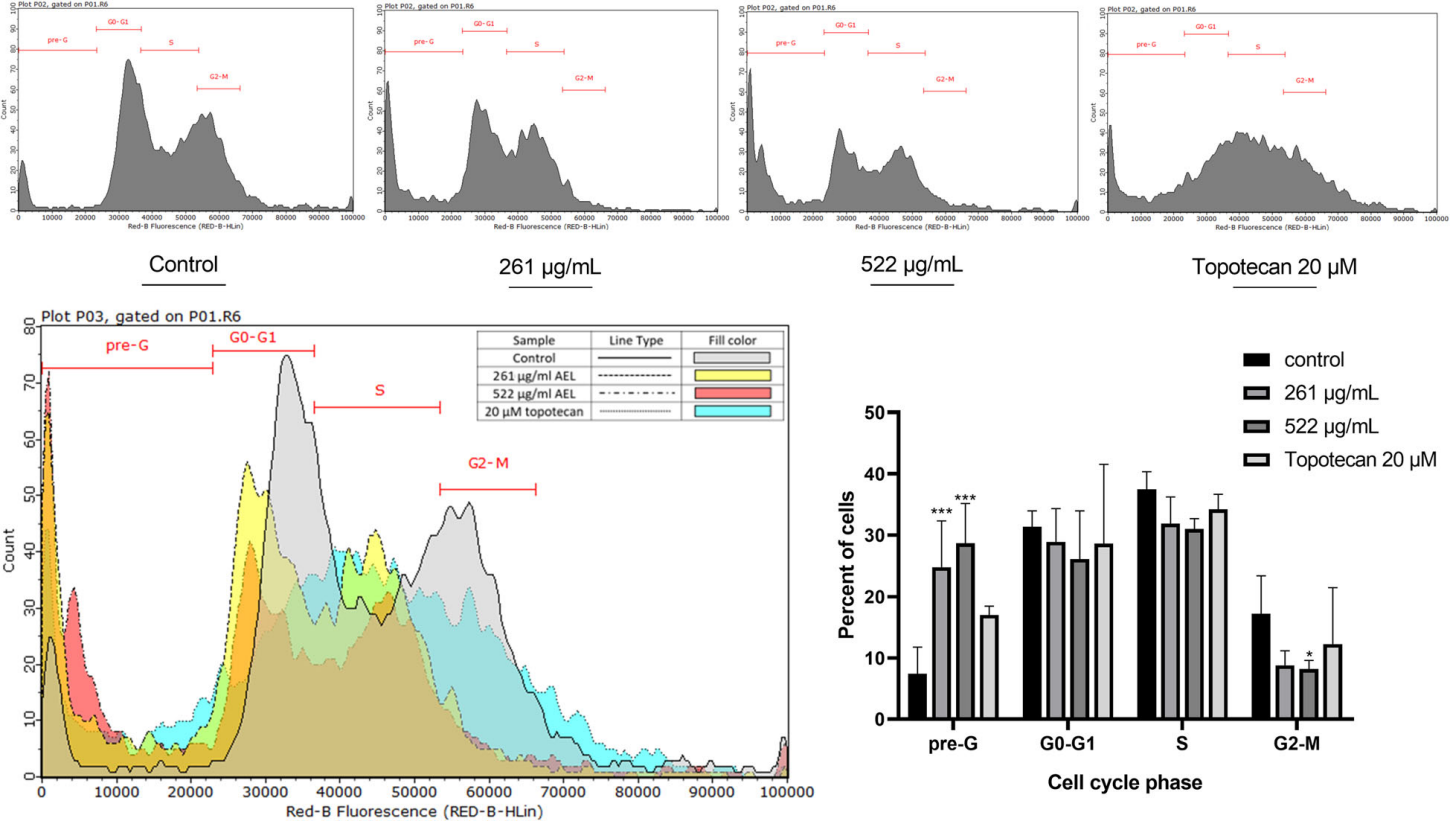
The effect of AELE on DNA fragmentation in MDA-MB-231 cells using cell cycle analysis. Cell cycle analysis of MDA-MB-231 cells treated with increasing concentrations of AELE and a positive control treated with topotecan for 24 h, shows a dose-dependent increase in the pre-G_0_/G_1_ stage. Dunnett’s test was used to compare the different concentrations to the control. Two-way ANOVA was used to determine the significant differences reported with * indicating a *p*-value: 0.01 < *p* < 0.05, ** indicating a *p*- value: 0.001 < *p* < 0.01, and *** indicating a *p*-value: *p* < 0.001

The effect of AELE on the cell cycle and the DNA content of MDA-MB-231 cells was elucidated by PI staining followed by flow cytometry. A dose-dependent shift of MDA-MB-231 cells from the G0/G1, S, and G2/M to the pre-G0/G1 stage, was detected, where cells are fragmented and contain DNA <2n, after treatment of MDA-MB-231 with 261 μg/mL and 522 μg/mL of AELE for 24 h, the values within which the IC_50_ falls. The proportion of MDA-MB-231 cells in the pre-G0/G1 stage significantly increased from 7.42% in the untreated cells to 24.75 and 28.65% in cells treated with 261 μg/mL (before IC_50_) and 522 μg/mL (after IC_50_) respectively, and reached 17% upon treatment with 20 μM of topotecan (Fig. 2). This suggests that AELE leads to cellular fragmentation, rather than a cell cycle arrest.

### The effect of *A. cherimola* ethanolic leaf extracts on the induction of apoptosis

**Fig. 3:**
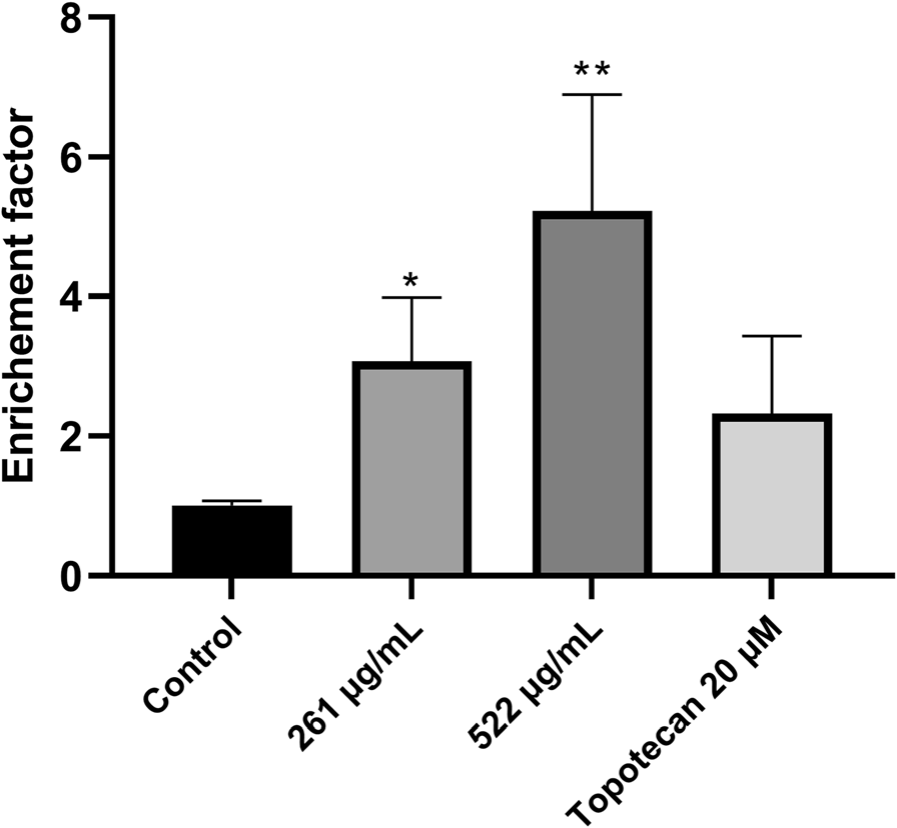
The quantitative effect of AELE on induction of apoptosis using Cell Death ELISA. A significant increase in DNA fragmentation is reported in MDA-MB-231 cells after 24 h of AELE treatment and topotecan which was used as a positive control. Plot 03 displays an overlay of the different treatments used (control in grey, 261 μg/mL in yellow, 522 μg/mL in pink, and the positive control in blue). Dunnett’s test was used to compare the different concentrations to the control. Ordinary one-way ANOVA was used to determine the significant differences reported with * indicating a *p*-value: 0.01 < *p* < 0.05, ** indicating a *p*- value: 0.001 < *p* < 0.01, and *** indicating a *p*-value: *p* < 0.001

To further elucidate the mechanism by which AELE exhibited its cytotoxic and cellular fragmentation effects, we quantified DNA fragmentation, which is a major hallmark of apoptosis, using Cell Death Detection ELISA. The enrichment factor reported is the ratio of the absorbance measured for each treatment concentration to that of the untreated control and indicates the abundance of cytosolic nucleosomes in the cells. A 3.07 and 5.22-fold increase in the enrichment factor was observed in MDA-MB-231 cells treated with 261 μg/mL and 522 μg/mL for 24 h, respectively. This enrichment factor reached 2.32 upon treatment with 20 μM topotecan (Fig. 3).

**Fig. 4:**
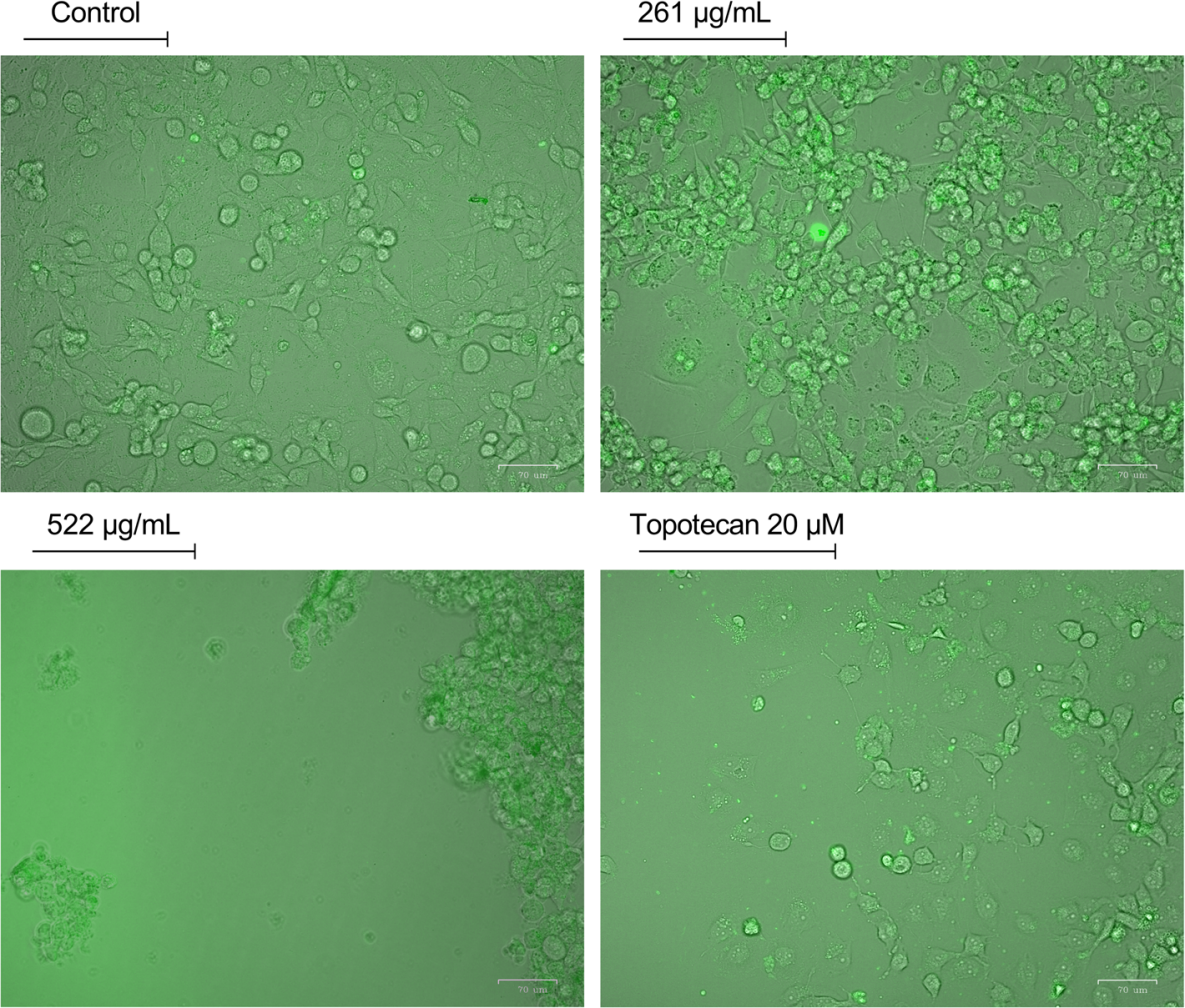
The qualitative assessment of apoptosis induced by AELE using Annexin V staining. Annexin V staining of MDA-MB-231 cells treated for 24 h with increasing concentrations of AELE and a positive control treated with topotecan shows a gradual increase in the number of Annexin-positive cells between the control group and the treated cells

To further confirm the increase in apoptosis, Annexin V staining was performed, detected by fluorescent microscopy. Upon treating MDA-MB-231 cells with increasing concentrations of AELE and topotecan for 24 h, a marked increase in Annexin V binding to the cell membrane was observed, which is concomitant with the flipping of phosphatidylserine moieties from the inner leaflet to the outer leaflet of the cell membrane, another major apoptotic hallmark (Fig. 4).

**Fig. 5:**
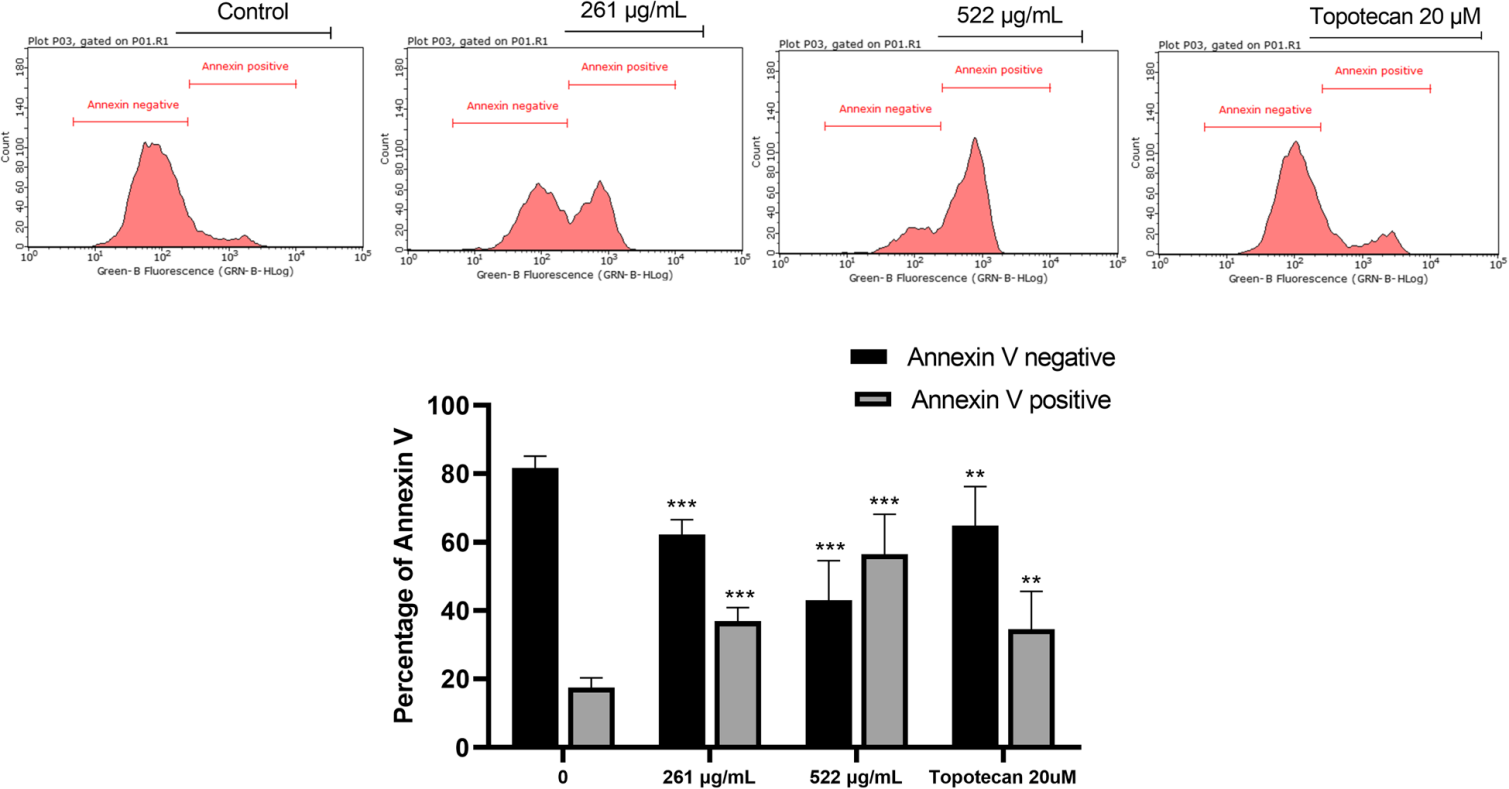
Flow cytometric analysis of Annexin V staining of MDA-MB-231 cells treated with AELE. Topotecan was used as a positive control. A significant increase in Annexin-V binding was reported after 24 h of treatment with increasing AELE concentrations. Dunnett’s test was used to compare the different concentrations to the control. Two-way ANOVA was used to determine the significant differences reported with * indicating a *p*-value: 0.01 < *p* < 0.05, ** indicating a *p*- value: 0.001 < *p* < 0.01, and *** indicating a *p*-value: *p* < 0.001

Annexin V staining and visualization by microscopy was followed by a quantitative assessment of Annexin V binding using flow cytometry. After 24 h of treatment with increasing concentrations of AELE, a significant dose-dependent increase in Annexin V positive cells was observed, from 17.43% in untreated cells to 36.85 and 56.50% Annexin V positive cells, paralleled with a decrease in Annexin V negative cells, from 81.64% in untreated cells, to 62.33 and 43.07% Annexin V negative cells, in MDA-MB-231 cells treated with 261 μg/mL and 522 μg/mL of AELE, respectively. Treatment of MDA-MB-231 cells with 20 μM of topotecan for 24 h showed a 34.56% Annexin V positive cells and 64.86% Annexin V negative cells, as compared to the control untreated cells. (Fig. 5). The increase in fluorescence using qualitative and quantitative Annexin V staining, accompanied by an increase in DNA fragmentation confirms that AELE induces apoptosis in the MDA-MB-231 cell line in a dose-dependent manner.

### The effect of *A. cherimola* leaf extracts on the expression of proteins involved in proliferative and apoptotic pathways

**Fig. 6:**
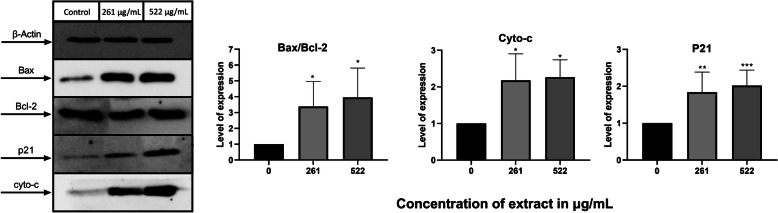
The effect of AELE on the expression of pro- and anti-apoptotic proteins. The results of Western Blot analysis of apoptosis-regulating proteins in MDA-MB-231 cells upon AELE treatment show an increased Bax-Bcl2 ratio, and upregulated pro-apoptotic proteins cytochrome c and p21. Dunnett’s test was used to compare the different concentrations to the control. Ordinary one-way ANOVA was used to determine the significant differences reported with * indicating a *p*-value: 0.01 < *p* < 0.05, ** indicating a *p*- value: 0.001 < *p* < 0.01, and *** indicating a *p*-value: *p* < 0.001

Western Blot analysis was performed to further elucidate the mechanism by which the anti-proliferative and pro-apoptotic pathways were induced. The effect of AELE on the expression of the cyclin-dependent kinase inhibitor 1 p21, cytochrome c, the pro-apoptotic protein Bax and the anti-apoptotic protein Bcl2 were assessed. A significant upregulation of p21 and cytochrome c was observed, compared to control untreated cells. Moreover, a significant increase in the Bax /Bcl2 ratio revealed a Bax/Bcl2 dependent pathway for apoptosis induction (Fig. 6). These results confirm the activation of the mitochondrial apoptotic pathway upon AELE treatment.

## Discussion

Herbs and plants have been widely consumed for their bioactive compounds and physiological benefits [44]. They can be used in different ways including teas, syrup, ointment, and tablets [45]. Alternative medicines from natural products have been extensively investigated and proved to be effective in treating cancer [46] due to the development of adverse effects resulting from chemotherapy [47].

Most research conducted on different closely related *Annona* species like *A. muricata* and *A. squamosa*, has focused on their anti-cancer properties against different cancer cell lines [48, 49]. Moreover, *A. cherimola* leaves are frequently sold and consumed by people to improve their health [50]. Recent studies revealed the anti-proliferative effect of *A. cherimola* leaves [21] and seeds [19] on leukemic cells. In the current study, the ethanolic leaf extract of *A. cherimola* was examined on breast cancer cell lines, namely MDA-MB-231 and MCF-7.

The results reported suggest a selective antiproliferative effect in a time- and dose-dependent manner on MDA-MB-231, with an IC_50_ of 555.3 μg/mL and 390.2 μg/mL at 24 and 48 h, respectively. These concentrations are lower than the concentrations found in a previous study of an ethanolic extract from *Urtica membranacea* having a potent anti-cancer effect at 750 μg/mL and 1500 μg/mL, which was efficiently correlated to mice breast cancer model treatment with no side effects [51].

Moreover, MSCs from the rat bone marrow, treated after adherence to mimic the attached breast cancer cells, did not reveal any cytotoxic effect of the extract; this further confirms the safety of this extract, which we have recently reported to exhibit no cytotoxic effects on normal mononuclear cells from human bone marrow [21]. Collectively, these results indicate the selective effect of AELE in targeting cancerous cells with no harm to normal healthy cells.

To explain the mechanism behind this anti-proliferative effect, the rest of the experiments were performed after 24 h of treatment; the results confirmed nuclear and membranes changes, which are two important hallmarks of apoptosis. A dose-dependent increase in apoptosis between control and treated cells was detected in the translocation of the phosphatidylserine to the outer leaflet and the increase in Annexin V binding. This was similar to another study conducted on the leaves of a closely related species *A. muricata,* which reported cytotoxic effects on breast cancer cell lines supported by the increase in Annexin V binding [21, 48]. Additionally, an increase in DNA fragmentation was detected using Cell Death ELISA, in addition to cellular fragmentation as revealed by cell cycle analysis using flow cytometry, whereby a significant increase in the pre-G_0_ phase (DNA < 2n) was reported. This could explain the presence of damaged cells or fragmented cell portions, in favor of the pro-apoptotic effect of the extract being investigated.

Western blot analysis was performed to further elucidate the AELE induced-apoptosis, where the upregulation of the pro-apoptotic Bax, cytochrome-c, and p21 in addition to the downregulation of anti-apoptotic Bcl-2 protein were detected. This mechanism was found to be induced via the mitochondrial pathway supported by the increase of the Bax/Bcl-2 ratio upon exposure of the cells to the extract. The overexpression of p21 can induce the expression of Bax [52]; moreover, insertion of Bax proteins into the mitochondrial membrane increases its permeability, leading to the release of cytochrome c which is followed by a cascade of protein activation leading to apoptotic cell death [53, 54]. Also, they will cause DNA damage and fragmentation which was confirmed through the significant increase in DNA fragmentation detected in Cell Death ELISA [55]. Previous studies have similarly reported that the pro-apoptotic effect of the leaves of the closely related *A. muricata* species occurs through upregulation of Bax, downregulation of Bcl-2 and cytochrome c leakage from the mitochondria [56, 57].

The extract used in this study has been previously analyzed in our laboratory, and its composition was found to be rich in Terpinolene (16.0619%), Germacrene D (15.2476%), and Alpha-tocopherol (15.0038%) [21]. D-limonene and carvacrol, of the terpene family, showed to induce apoptosis through the mitochondrial pathway [58, 59], similar to the results observed in our study. Another compound, Germacrene D, was found to exhibit a cytotoxic effect on HL-60 cells [60] and human cervical, liver, and hepatocellular carcinoma [61]. Alpha-tocopherol derivatives, another major constituent, has been found to induce cell death and inhibit viability, migration, and invasion of breast cancer cells [62].

As discussed previously, the AELE was found to exhibit a selective pro-apoptotic and anti-proliferative effect on MDA-MB-231 cells. A similar study by BG Utage et al., investigated the methanolic leaf extract of *Prosopis juliflora* on breast cancer cell lines. This extract was found to be rich in bioactive compounds such as terpenes and phytol, similar to the major compound detected in AELE. They revealed the efficient and selective anti-breast cancer activity against triple negative breast cancer cells (MDA-MB-231) [63], explaining the selective effect observed in our study.

## Conclusions

*Annona cherimola* ethanolic leaf extract, previously found to exhibit an anti-proliferative and pro-apoptotic effect on AML cell line, can also induce apoptosis in a dose-and time-dependent manner exclusively on the triple-negative breast cancer cell line, MDA-MB-231. This mechanism was found to be through the mitochondrial pathway by releasing cytochrome c, upregulated p21 and increasing Bax/Bcl-2 ratio. Future work aims at fractionating the extract to identify the bioactive compounds responsible for the effects observed on MDA-MB-231 and to further examine its efficacy in vivo.

## Data Availability

All data generated or analyzed during this study are included in this published article (and its supplementary information files).

## Abbreviations

AELE: Annona ethanolic leaf extract
AML: Acute myeloid leukemia
DMEM: Dulbecco’s Modified Eagle Medium
ER: Estrogen receptor
FBS: Fetal Bovine Serum
GC-MS: Gas Chromatography-Mass Spectrometry
IC50: Half-maximal inhibitory concentration
MSCs: Mesenchymal stem cells
PBS: Phosphate Buffered Saline
PI: Propidium Iodine
PR: Progesterone receptor
SEM: Standard Error of the mean
